# The use of centrifugated autologous lipoaspirate in the endoscopic management of idiopathic subglottic stenosis: results from a retrospective non-randomized study

**DOI:** 10.1007/s00405-026-10322-9

**Published:** 2026-06-01

**Authors:** Francesco Mattioli, Margherita Basso, Antonio Moretti, Greta Morselli, Caterina Vaccari, Edoardo Serafini, Cinzia Del Giovane, Massimo Pinelli, Daniele Marchioni, Alessandro Marchioni

**Affiliations:** 1 Department of Otorhinolaryngology-Head and Neck Surgery, University Hospital of Modena, Largo del Pozzo, 71, Modena, 41124 Italy; 2 Respiratory Diseases Unit and Center for Rare Lung Disease, Department of Surgical and Medical Sciences, University Hospital of Modena, Modena, Italy; 3 Department of Otorhinolaryngology-Head and Neck Surgery, University Hospital Sant’Orsola of Bologna, Bologna, Italy; 4 Unit of Statistical and Methodological Support to Clinical Research, University Hospital of Modena, Modena, Italy; 5 Institute of Primary Health Care (BIHAM), University of Bern, Bern, Switzerland; 6 Department of Medical and Surgical Sciences, Division of Plastic Surgery, University of Modena and Reggio Emilia, Policlinico of Modena, Modena, Italy

**Keywords:** Idiopatic subglottic stenosis, Laryngotracheal stenosis, Regenerative medicine, Autologous lipoaspirate injection

## Abstract

**Purpose:**

Idiopathic subglottic stenosis (iSGS) is a rare, progressive fibroinflammatory disease that predominantly affects middle-aged women. Although endoscopic treatments have improved, recurrence rates remain high and no standardized therapeutic approach has been defined. This exploratory retrospective pilot study aimed to assess feasibility, safety and explore the preliminary signal of benefit of intralesional injection of centrifuged autologous lipoaspirate as an adjunct to endoscopic balloon dilation in patients with iSGS.

**Methods:**

A retrospective pilot observational study was conducted at the Tracheal Team of the University Hospital of M. and the Otorhinolaryngology Unit of S.O. Hospital, B., I., from 2015 to 2024. Twenty-six adult women with histologically confirmed iSGS and at least 12 months of follow-up were included. Patients received either standard endoscopic balloon dilation with intralesional corticosteroid injection (n=19) or balloon dilation followed by injection of centrifuged autologous lipoaspirate (n=7). The primary outcome was recurrence within 24 months, defined as the need for reintervention due to symptomatic restenosis. Secondary outcomes included time-to-recurrence, Subglottic Stenosis–6 (SGS-6) scores, and perioperative complications.

**Results:**

Overall, recurrence occurred in 16 of 26 patients (61.6%). Recurrence was lower in the lipoaspirate group (2/7, 28.6%) compared with the standard group (14/19, 73.7%), with Pearson’s χ² of 4.4 (P = 0.036; Fisher’s exact P=0.06). Median time-to-recurrence was longer in the lipoaspirate group (482 days) than in the standard group (240 days) (Long-rank test p=0.047).

**Conclusions:**

In this retrospective pilot study, patients with idiopathic subglottic stenosis treated with intralesional injection of centrifuged autologous lipoaspirate following endoscopic balloon dilation showed a preliminary signal of lower recurrence rate and longer recurrence-free survival.

**Graphical abstract:**

**Figure:**
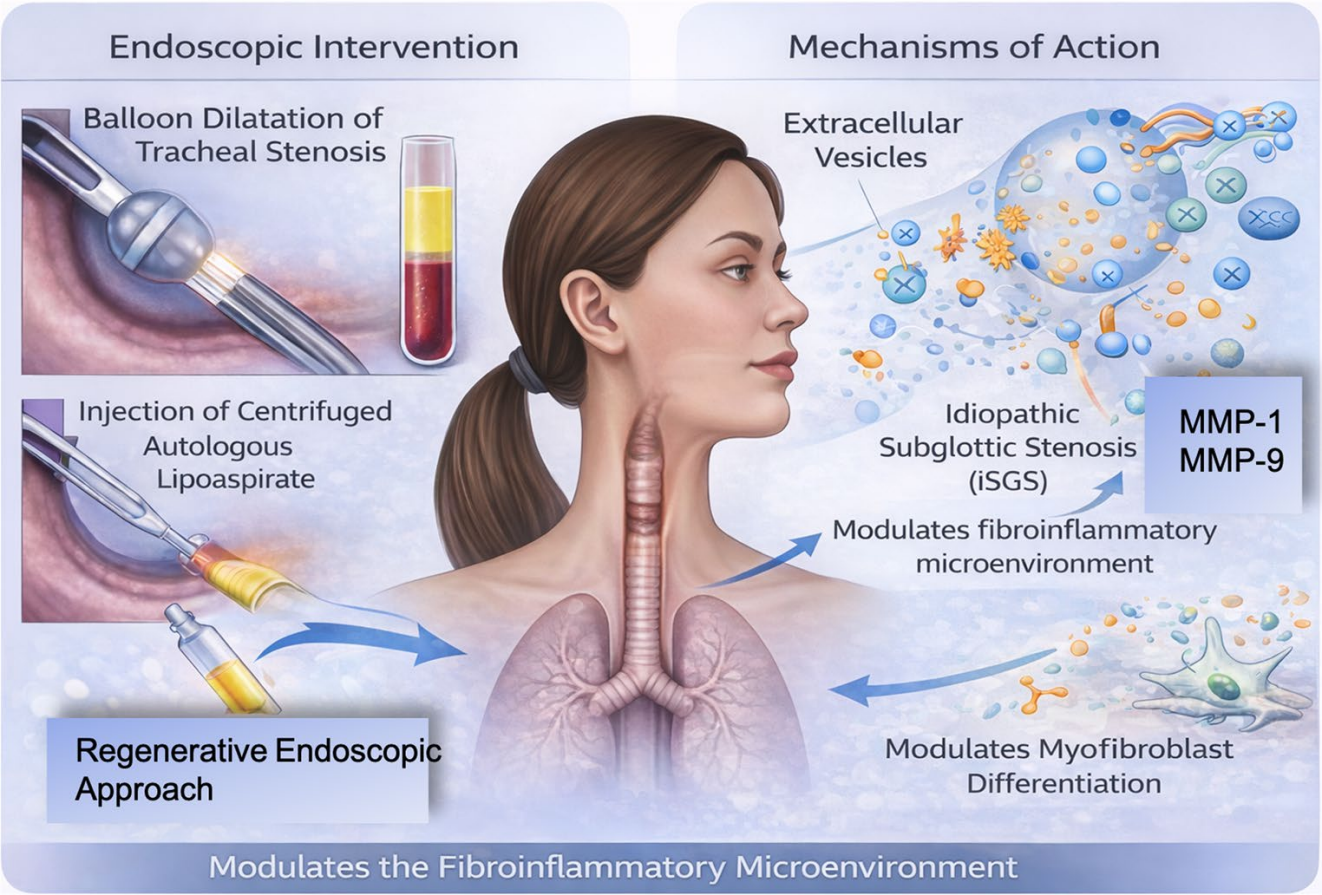

## Key points

Question: Is there an indication of reduction in the recurrence in patients with iSGS treated with intralesional injection of centrifuged autologous lipoaspirate? Is the procedure feasible and safe as an adjunct to endoscopic balloon dilatation?

Findings: In this retrospective pilot study of 26 women with iSGS, treatment with balloon dilation followed by lipoaspirate injection showed a lower observed recurrence rate (28.6% vs 73.7%) and a longer time-to-recurrence (482 vs 240 days) compared with standard corticosteroid injection. The procedure was feasible and safe for all patients with no peri-operative complications.

Meaning: Those preliminary findings suggest that centrifuged autologous lipoaspirate may be a promising, biologically active adjunct to endoscopic therapy for iSGS, and seems to support the need for prospective controlled studies to further evaluate its clinical role.

## Introduction

Idiopathic subglottic stenosis (iSGS) is a rare fibro-inflammatory disease characterized by progressive concentric narrowing of the airway at the level of the cricoid and first tracheal ring, occurring almost exclusively in middle-aged women [1–3]. Although traditionally considered a cicatricial disorder of unclear etiology, recent molecular investigations have revealed that iSGS represents a complex immune-mediated mucosal disease driven by dysregulated inflammation, aberrant epithelial repair, and maladaptive extracellular matrix (ECM) deposition. Clinical presentation is frequently misleading, and patients are often misdiagnosed with asthma or COPD for years before receiving the correct diagnosis [4, 5].

Growing evidence indicates that iSGS arises from an aberrant T cell–mediated immune response within the subglottic mucosa. Histological samples consistently show increased infiltration of CD4 + and CD8 + T lymphocytes, accompanied by heightened expression of inflammatory cytokines. Pathologic fibroblasts derived from iSGS tissue exhibit exaggerated responsiveness to these signals, amplifying inflammation and promoting excessive ECM deposition [6, 7]. Central to this response is the induction of epithelial–mesenchymal transition (EMT), in which respiratory epithelial cells adopt a mesenchymal phenotype, contributing to fibroblast expansion and collagen accumulation [8]. Dysregulated EMT is driven by multiple cytokine networks, particularly the IL-23/IL-17 axis, which is significantly upregulated in iSGS tissue and promotes fibroblast proliferation, collagen deposition, and persistent recruitment of inflammatory cells [9, 10].

Another critical pathway involves the programmed death-1/programmed death-ligand 1 (PD-1/PD-L1) immune checkpoint, whose overactivation contributes to chronic T-cell exhaustion and ineffective resolution of inflammation, a mechanism shared with idiopathic pulmonary fibrosis and other chronic fibrotic disorders [11]. Additional mediators such as IL-6, IFN-γ, CCL2, and antimicrobial γδ T-cells underscore the presence of a persistent, dysregulated immune environment, likely triggered by host–microbe interactions. Indeed, recent profiling of subglottic mucosa suggests that even in the absence of overt infection, a low-grade microbial stimulus—often involving species such as Moraxella and Acinetobacter—may activate innate and adaptive immune pathways, sustaining inflammation below histological detection thresholds [12, 13].

Current management of iSGS remains challenging. Endoscopic techniques—such as balloon dilation with or without intralesional corticosteroids or mitomycin C—are the mainstay for mild to moderate stenosis but are limited by high recurrence rates, often exceeding 70% [14, 15]. Open surgical procedures (cricotracheal resection or laryngotracheal reconstruction) offer longer disease-free intervals but are associated with greater morbidity and potential voice changes. This has fostered interest in biologically active, regenerative therapies capable of modulating the inflammatory microenvironment and promoting physiological mucosal repair [16].

Autologous adipose tissue represents a particularly promising candidate in this setting. Centrifuged lipoaspirate, enriched in adipose-derived mesenchymal stem/stromal cells (AD-MSCs), stromal vascular fraction (SVF), and extracellular vesicles (EVs), exhibits potent immunomodulatory, anti-fibrotic, and pro-regenerative properties [17–19]. AD-MSCs secrete regulatory cytokines—including IL-10, TGF-β1, VEGF, PGE₂, and nitric oxide—that inhibit T-cell activation, downregulate fibroblast activity, and enhance epithelial healing [20–22]. Preclinical models of subglottic and tracheal injury have demonstrated that adipose-derived cell therapies reduce fibrosis, suppress pro-inflammatory cytokines (IL-1α, IL-6, TNF-α), and restore normal airway architecture [23, 24].

Based on this rationale, the Tracheal Team of the University Hospital of M. introduced the use of centrifuged autologous lipoaspirate as an adjunct to endoscopic dilation for iSGS in 2021. The aim of this exploratory retrospective pilot study was to explore whether intralesional injection of centrifuged autologous lipoaspirate is associated with preliminary signal of longer time-to-recurrence compared with standard endoscopic treatment and therefore could be evaluated as an adjunct to endoscopic ballon dilatation in iSGS.

## Methods

### Study design and setting

This retrospective pilot observational study included consecutive patients treated for idiopathic subglottic stenosis at two tertiary centers—the University Hospital of M. and S.O Hospital in B., I.—between January 1, 2015, and August 31, 2024. This research was conducted in full accordance with the World Medical Association Declaration of Helsinki (2002). The study was approved by the local institutional ethics committees. Written informed consent for the publication of anonymized clinical images was obtained.

### Participants

Eligible patients were adults (≥ 18 years) with histologically confirmed idiopathic subglottic stenosis, negative autoimmune serology, and at least 12 months of follow-up after endoscopic intervention. Exclusion criteria included active infection, autoimmune disease, or previous open airway reconstruction. Twenty-six patients met inclusion criteria. All were female, consistent with the known demographic profile of iSGS. Median age at diagnosis was 53 years (IQR, 48–59). Comorbidities were minimal, with a median Charlson Comorbidity Index (CCI) of 1 (IQR, 0–1).

### Interventions

Two treatment groups were defined according to the treatment chosen by the attending physician:

1. Standard group (*n* = 19): Endoscopic balloon dilation followed by intralesional corticosteroid injection (acetonide triamcinolone).
2. Lipoaspirate group (*n* = 7): Endoscopic balloon dilation followed by intralesional injection of centrifuged autologous lipoaspirate.

Patients selected for balloon dilation and triamcinolone injection were at their first intervention for subglottic stenosis and underwent a concomitant biopsy of the stenotic mucosa for diagnostic assessment.

Lipoaspirate injection was preferentially offered to patients with relapsing disease who had previously received only endoscopic treatments, and prior histologic confirmation of idiopathic etiology (biopsy negative for vasculitis or storage disorders), in keeping with the Regenerative Protocol adopted by the Modena Tracheal Team since 2021. Of these, five were at their second overall treatment, and two had undergone four previous endoscopic procedures.

All procedures were performed under general anesthesia with spontaneous ventilation. A Lindholm laryngoscope^®^ was used for exposure. Balloon dilation (Aeris Tracoe^®^ 8–10 mm) was performed at 10 atmospheres for 30 s. In the lipoaspirate group, adipose tissue was harvested from the lower abdomen, centrifuged at 3000 rpm for 3 min using the ELEA^®^ system (Waldner Medical Technologies), and injected trans-orally into the stenotic segment using an 18-gauge needle. Multiple small-volume injections were distributed circumferentially within the fibrotic tissue. Patients were discharged after 24 h and followed up at 2 and 5 months with flexible laryngoscopy and SGS-6 questionnaire assessment [1].

### Outcome measures

**Fig. 1:**
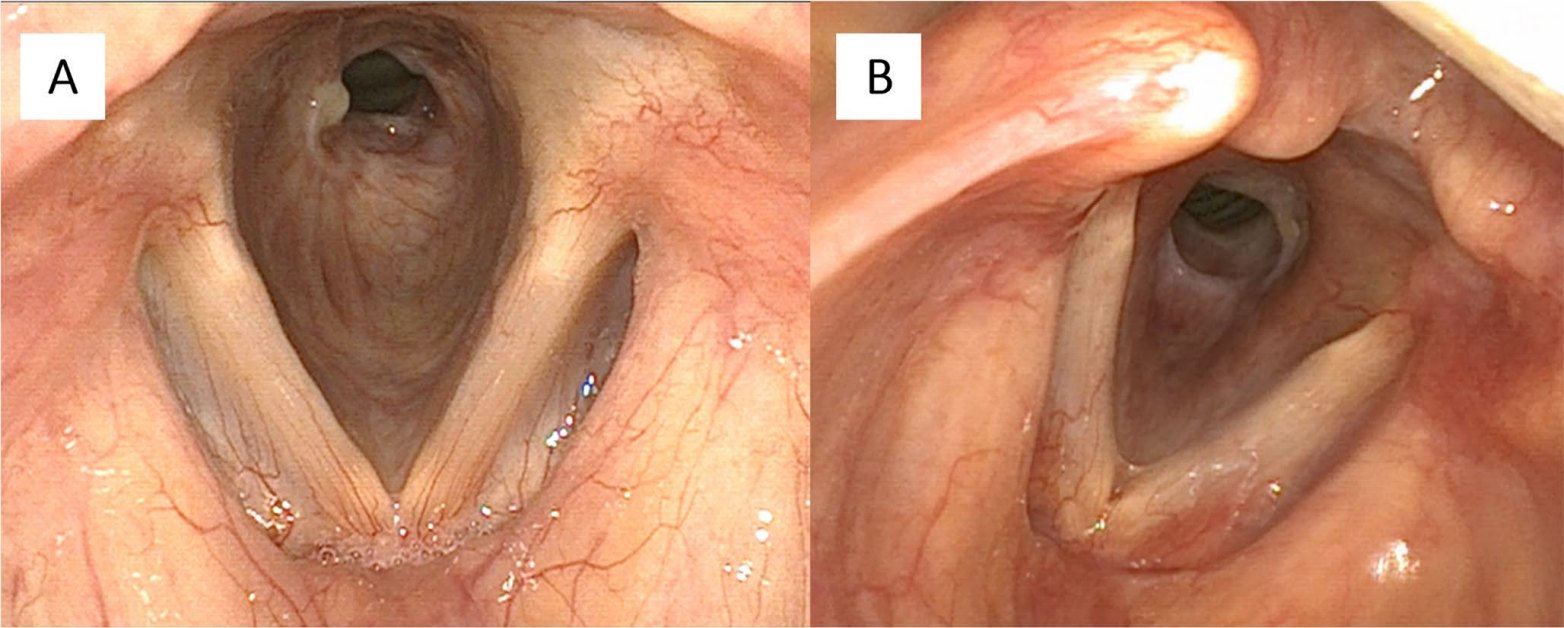
**A**: Idiopathic subglottic stenosis during an active inflammatory phase, with evi-dent granulation and hyperemia. **B**: Idiopathic subglottic stenosis 30 days after injection of centrifuged lipoaspirate, showing absence of hyperemia at the stenotic segment

The primary outcome was recurrence over 24 months, defined as the need for repeat endoscopic intervention due to symptomatic restenosis (Fig. 1). Secondary outcomes included: time-to-recurrence, SGS-6 symptom score, perioperative complications. Stenosis severity was classified according to the Cotton-Myer and McCaffrey staging system, as detailed in Table 1.

**Table 1:** Baseline demographic and clinical characteristics. CCI: Charlson Comorbidity Index; CM: Cotton-Myer

	Total*n* 26 = (%)	Ballon + corticosteroids*n* 19= (73)	Ballon + lipoaspirate*n* 7= (27)
Age, median at diagnosis, years (IQR)	53 (11)	54 (12)	53 (14)
Sex, (%) M F	0 (0) 26 (100)	0 (0) 19 (100)	0 (0) 7 (100)
Comorbidities, n (%) Yes No CCI, median at diagnosis (IQR)	5 (19) 21 (81) 1 (1)	5 (26) 14 (74) 1 (2)	0 (0) 7 (100) 0.5 (1)
CM classification I II III IV	2 (8%) 11 (42%) 11 (42%) 2 (8%)	1 (5) 7 (36) 9 (44) 3 (15)	1 (14) 4 (57) 2 (29) 0 (0)
McCaffrey classification I II III	20 (76.9) 4 (15.4) 2 (7.7)	15 (78.9) 4 (21.1) 0 (0)	5 (71.4) 0 (0) 2 (28.6)
BMI, median at diagnosis (IQR)	21.45 (4.3)	22.5 (5.1)	20.7 (4.3)
At rest dyspnea, n (%) Yes No	8 (31) 18 (69)	7 (36) 14 (64)	1 (14) 6 (86)
Exertional dyspnea, n (%) Yes No	26 (100) 0 (0)	29 (100) 0 (0)	7 (100) 0 (0)
Wheezing, n (%) Yes No	4 (15) 22 (85)	3 (15) 16 (85)	1 (14) 6 (86)
Acute respiratory insufficiency, n (%) Yes No	3 (11) 23 (89)	3 (15) 16 (85)	0 (0) 7 (100)

### Statistical analysis

Descriptive statistics summarized demographic and clinical data. Continuous variables were expressed as median (IQR) and compared using the median test. Categorical variables were compared with Pearson χ² test or Fisher’s exact test. Odds ratios (ORs) and 95% confidence intervals (CIs) were calculated. Kaplan–Meier survival analysis estimated time-to-recurrence; differences between treatment groups were evaluated using the log-rank test. Statistical significance was set at *P* < 0.05. Analyses were performed using STATA v18 (StataCorp).

## Results

### Patient characteristics

Baseline demographic and clinical data are shown in Table 1 (Supplementary Material). All 26 patients were female, with similar age, body mass index, and comorbidity profiles between groups. No significant differences were observed in stenosis severity according to Cotton-Myer or McCaffrey classification. The most common presenting symptom was exertional dyspnea (100%), followed by rest dyspnea (31%) and wheezing (15%). No patient had autoimmune markers or systemic inflammatory disease.

### Procedural and postoperative outcomes

**Fig. 2:**
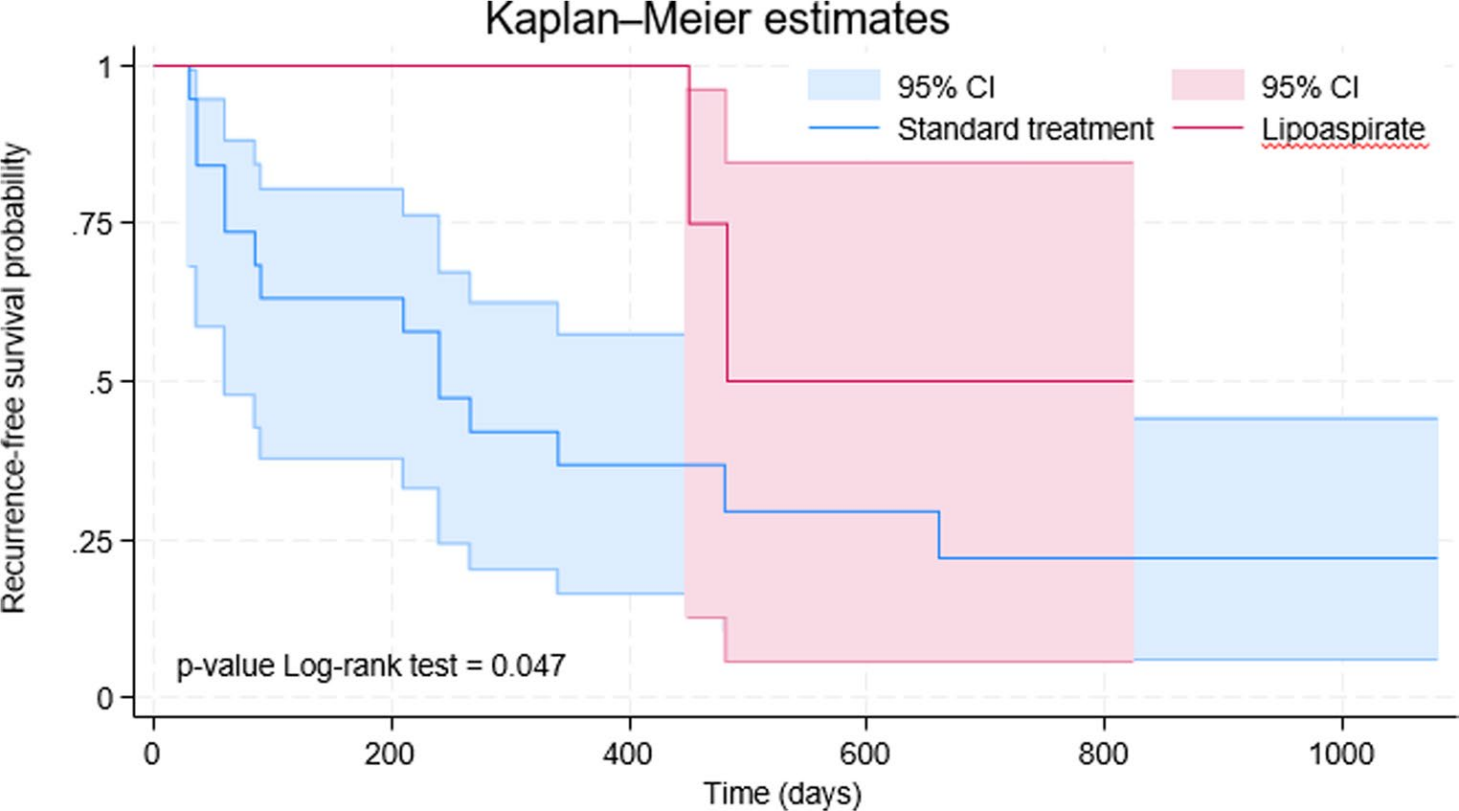
Kaplan–Meier recurrence-free survival curves for standard vs. lipoaspirate groups

Median hospital stay was 1.5 days (range, 1–3). No intraoperative or postoperative complications were observed, and lipoaspirate injection was feasible in all treated patients. Within 12 months, recurrence occurred in 12 of 26 patients (46.2%), and within 24 months, in 16 of 26 (61.6%). Recurrence events differed between groups: lipoaspirate group, 2 of 7 (28.6%); standard group, 14 of 19 (73.7%) (Pearson χ² test *P* = 0.036; Fisher’s exact *P* = 0.06). The odds ratio suggested a possible protective effect of the lipoaspirate (OR = 0.14); however, the estimate was imprecise, as reflected by the wide 95% confidence interval (0.01–1.32). Median time-to-recurrence was significantly longer in the lipoaspirate group (482 days) than in the standard group (240 days; *P* = 0.047) (Fig. 2). At the last follow-up, only 3 patients in the standard group reported persistent exertional dyspnea, whereas all lipoaspirate-treated patients were asymptomatic.

### Patient-reported outcomes

The SGS-6 questionnaire was administered to 10 patients from 2019 onward during the follow-up. Median SGS-6 score was 9 in the lipoaspirate group versus 4 in the standard group, with no association between SGS-6 score and recurrence (*P* = 0.19).

## Discussion

In this exploratory retrospective pilot study, intralesional injection of centrifuged autologous lipoaspirate as an adjunct to endoscopic balloon dilation was associated with a preliminary signal of longer time-to-recurrence compared with standard intralesional corticosteroid injection in idiopathic subglottic stenosis. These preliminary findings cautiously suggest a potential clinical benefit that is consistent with current insights into the molecular pathophysiology of iSGS and the known biologic activities of adipose-derived regenerative therapies, however these results need to be interpreted with caution given the small sample size and retrospective design of the study. Recurrence rates after endoscopic dilation in iSGS are notoriously high, with published rates ranging from 40% to 100%, and mean recurrence-free intervals of 8–12 months [15, 25]. In our study formed of a small sample, the recurrence rate of 73.7% in the standard group aligns with literature, whereas the 28.6% rate in the lipoaspirate group represents a meaningful reduction. The median time-to-recurrence interval of 482 days also exceeds typical outcomes reported for steroid-based protocols [3, 26, 27]. The decision to perform reintervention was always made collegially by the Tracheal Team, thereby reducing potential biases related to the clinical behavior of individual specialists. It is worth noting that patients in the lipoaspirate group had undergone multiple previous endoscopic procedures, and were therefore selected in a non-randomized manner, which introduces potential confounding by indication and selection bias. Patients with relapsing disease are generally more challenging to treat because of progressive tissue remodeling and increased fibrotic stiffness, and this imbalance may have influenced the observed outcomes, underestimating them, thus, these results should be interpreted with caution. Nevertheless, the observation of a favorable signal in this clinically complex population highlights the need for prospective controlled studies to clarify whether lipoaspirate injection may offer a true clinical benefit. The apparently counter-trend result of the SGS-6 questionnaire may find a possible explanation in the temporal bias with which it was administered to patients, namely from 2019 onward. Further studies are needed to validate its effectiveness in identifying the approach of a recurrence of idiopathic subglottic stenosis.

I-SGS is increasingly recognized as an immune-driven fibroinflammatory disorder, rather than purely cicatricial stenosis. In iSGS dysregulated inflammatory signaling promotes activation, extracellular matrix deposition, and impaired epithelial repair, creating a microenvironment prone to recurrent fibrosis and restenosis [5]. Preclinical and translational studies suggest that adipose-derived regenerative therapies may modulate this process by downregulating proinflammatory cytokines activity and supporting epithelial healing and tissue remodeling [11, 22, 28]. Centrifugation of lipoaspirate further concentrates the biologically active cellular and stromal fraction while removing oil and debris, potentially enhancing the regenerative and immunomodulatory effects [29, 30]. ELEA^®^ (Surgere) is an operator-independent medical device that applies orbital mechanical forces to adipose tissue, promoting cellular concentration without micro-fragmentation. This process preserves tissue integrity while enhancing its biological activity. Treated tissue shows increased expression of TSG-6 and upregulation of pluripotency-related transcription factors (Sox2, OCT4, Nanog), associated with regenerative potential. The ability to perform multiple small injections circumferentially within the stenotic segment likely maximizes tissue exposure to these bioactive components, promoting uniform modulation of the local fibroinflammatory milieu [31].

Together, these mechanisms provide a biologically plausible rationale for the exploratory clinical signal observed in this pilot study, while underscoring the need for prospective studies to clarify the therapeutic role of lipoaspirate in iSGS. In particular, the potential benefit suggested by our study may arise from multiple mechanisms: (1) immunomodulation—ADSCs suppress T-cell proliferation and induce regulatory T-cell activation, mitigating chronic inflammation; (2) antifibrotic action—downregulation of myofibroblast activation and reduced extracellular matrix deposition may limit restenosis; (3) regenerative support—the adipose micro-environment promotes neovascularization and epithelial regeneration, improving tissue compliance and healing [20, 22, 32]. For clinicians, the integration of centrifuged lipoaspirate into endoscopic protocols could represent a low-risk, autologous, and biologically active adjunct therapy. The absence of adverse events and the feasibility of cryopreserving residual lipoaspirate for future injections further enhance its practicality. Importantly, this approach aligns with the trend toward regenerative and minimally invasive management of airway diseases.

### Limitations and future directions

This study has several important limitations inherent to its retrospective and non-randomized design. First, treatment allocation was based on clinical judgment, with lipoaspirate injection preferentially offered to patients with relapsing disease, introducing potential confounding by indication and selection bias that may overestimate the apparent effect. Second, the two treatment groups were unbalanced in size and were treated over different time periods, which may have introduced time-period confounding and limit the interpretability of our comparative analyses. Third, the small sample size and small number of events, particularly in the lipoaspirate arm, limits the precision of the time-to-recurrence analysis and may overestimate the apparent effect. Fourth, recurrence was defined by the decision to reintervene, which may be influenced by clinical behavior, patient expectations, and the novelty of the treatment, and therefore represents a potentially subjective outcome. Fifth, patient-reported outcomes were incompletely available, and SGS-6 scores were collected only in more recent years, further limiting the ability to draw conclusions regarding symptomatic benefit. Finally, mechanistic pathways were not directly assessed in this study, and the biological rationale discussed remains speculative. Future prospective, multicenter controlled studies are needed to evaluate the feasibility, safety, and potential clinical benefit of lipoaspirate injection in idiopathic subglottic stenosis, ideally with standardized treatment protocols, predefined outcomes, and integrate biological correlations. Such studies will be essential to determine whether this regenerative approach offers a true therapeutic advantage over standard endoscopic management. Cytological characterization of the lipoaspirate represents another of the next steps of this study protocol, with the aim of describing its features and viability both at the time of harvest and after cryopreservation.

## Conclusions

In conclusion, preliminary results of this pilot study suggest a reduction of recurrence using intralesional injection of centrifuged autologous lipoaspirate when compared with standard endoscopic treatment, supporting its potential evaluation as an adjunct to endoscopic balloon dilatation in patients with iSGS. Furthermore, they showed a preliminary signal of longer time-to-recurrence compared with standard corticosteroid injection. However, given the non-randomized design, small sample size, and potential confounding by indication, these findings should be interpreted with caution. Large prospective controlled studies are needed to confirm feasibility and safety and to determine whether lipoaspirate injection provides a true clinical benefit over standard endoscopic management.

## Data Availability

The data underlying in this article are available within this article and in its supplementary materials. Further details can be provided by the corresponding author upon reasonable request.
